# CAREGIVER INVOLVEMENT AND CONCERNS WITH RESIDENT CARE IN ASSISTED LIVING BEFORE AND DURING COVID-19

**DOI:** 10.1093/geroni/igad104.1628

**Published:** 2023-12-21

**Authors:** Matthias Hoben, Hana Dampf, David Hogan, Kyle Corbett, Kim McGrail, Lauren Griffith, Andrea Gruneir, Colleen Maxwell

**Affiliations:** York University, Toronto, Ontario, Canada; University of Alberta, Edmonton, Alberta, Canada; Univeresity of Calgary, Calgary, Alberta, Canada; University of Alberta, Edmonton, Alberta, Canada; University of British Columbia, Vancouver, British Columbia, Canada; McMaster University, Hamilton, Ontario, Canada; University of Alberta, Edmonton, Alberta, Canada; University of Waterloo, Waterloo, Ontario, Canada

## Abstract

Family/friend caregivers are essential in promoting assisted living (AL) residents’ health/well-being, but their involvement was restricted during the COVID-19 pandemic. Care needs in AL are similarly complex as in nursing homes, but fewer staffing resources and services are available. Caregiver involvement and concerns with care of AL residents before and during the COVID-19 pandemic is a critical knowledge gap. This prospective cohort study collected online surveys from caregivers to AL residents in Western Canada. Surveys assessed socio-demographics, ways of visiting or communicating with residents, involvement in care tasks, concerns with resident physical/mental health, perceived lack of resident access to care services, and whether caregivers felt well informed/involved with resident care. Our first survey assessed outcomes in the three months before and after pandemic wave 1 (03/2020), our second survey assessed outcomes in pandemic wave 2 (11/2020 -02/2021). Based on 386 responses, in-person visits dropped significantly in wave 1 (91.5% to 30.6%) and so did caregiver involvement in nearly all care tasks (e.g., toileting: 7.5% to 2.6%, social/recreational activities: 73.6% to 25.7%). While these rates increased in wave 2, most did not return to pre-pandemic levels. Caregiver concerns increased in wave 1 (e.g., concerns about residents’ depressed mood: from 21.3% to 50.3%) and stayed high in wave 2. Concerns were particularly high (e.g., >20% higher for depressed mood) among caregivers who did not feel well informed/involved with resident care. Continued caregiver involvement in resident care and communication with caregivers is key to mitigating caregiver concerns and supporting caregiver and resident well-being.

